# Detecting flying insects using car nets and DNA metabarcoding

**DOI:** 10.1098/rsbl.2020.0833

**Published:** 2021-03-31

**Authors:** Cecilie S. Svenningsen, Tobias Guldberg Frøslev, Jesper Bladt, Lene Bruhn Pedersen, Jonas Colling Larsen, Rasmus Ejrnæs, Camilla Fløjgaard, Anders Johannes Hansen, Jacob Heilmann-Clausen, Robert R. Dunn, Anders P. Tøttrup

**Affiliations:** ^1^ Natural History Museum of Denmark, University of Copenhagen, Copenhagen, Denmark; ^2^ Section for GeoGenetics, GLOBE Institute, University of Copenhagen, Copenhagen, Denmark; ^3^ Centre for Macroecology, Evolution and Climate, GLOBE Institute, University of Copenhagen, Copenhagen, Denmark; ^4^ Department of Bioscience–Biodiversity and Conservation, Aarhus University, Aarhus, Denmark; ^5^ Department of Applied Ecology, North Carolina State University, Raleigh, NC, USA

**Keywords:** insect diversity, mass collected insects, DNA metabarcoding, COI, car nets, citizen science

## Abstract

Monitoring insects across space and time is challenging, due to their vast taxonomic and functional diversity. This study demonstrates how nets mounted on rooftops of cars (car nets) and DNA metabarcoding can be applied to sample flying insect richness and diversity across large spatial scales within a limited time period. During June 2018, 365 car net samples were collected by 151 volunteers during two daily time intervals on 218 routes in Denmark. Insect bulk samples were processed with a DNA metabarcoding protocol to estimate taxonomic composition, and the results were compared to known flying insect richness and occurrence data. Insect and hoverfly richness and diversity were assessed across biogeographic regions and dominant land cover types. We detected 15 out of 19 flying insect orders present in Denmark, with high proportions of especially Diptera compared to Danish estimates, and lower insect richness and diversity in urbanized areas. We detected 319 species not known for Denmark and 174 species assessed in the Danish Red List. Our results indicate that the methodology can assess the flying insect fauna at large spatial scales to a wide extent, but may be, like other methods, biased towards certain insect orders.

## 1. Introduction

Recent studies have highlighted declines in the biomass, abundance and diversity of insects [1–3]. These declines have come as a surprise, in part because of our poor understanding of spatial and temporal patterns in insect communities [3]. One reason for this dearth is logistic; insects collected via standardized sampling must be sorted manually. In biologically diverse regions, this is simply impossible, because most insect species have yet to be named [4] and thus, the results of sorting one sample are very difficult to compare to the results of another sample.

There exists a variety of sampling methods for insect surveying and their utilization depends on the scientific question addressed [5]. Since insects are the most speciose group of animals on Earth [4], no sampling method readily detects all taxa, e.g. Malaise traps disproportionately detect species from Diptera and Hymenoptera, two of the insect taxa most difficult to identify [6,7]. Most insect sampling methods are stationary or restricted in spatial scalability and can be difficult to use in landscapes in which most land is privately owned such as intensive agricultural fields and cities. Rooftop or fender car nets have the potential to sample in a way that integrates space [8,9] and have previously been used for targeted sampling of different types of flying insects, often with a focus on disease vectors, e.g. mosquitoes or black flies [10–17], or beetles [18–20]. Importantly, car nets can sample insects flying from both public and private lands, when the roads themselves are public.

### (a) DNA metabarcoding for diversity assessments

Here, we explore the addition of a novel innovation to car net sampling to overcome its chief existing barrier, sorting and identification. We seek to consider the potential of identifying the results of car net sampling through DNA metabarcoding techniques. The implementation of DNA metabarcoding techniques allows for fast and cost-effective processing of a large number of samples and can be used in monitoring programmes for community assessment [21]. With higher quality curated reference databases, large sample processing output and standardized monitoring schemes, DNA metabarcoding has the potential to become an applied method for insect diversity monitoring [22–24].

Our aim in this paper is to assess the car net sampling method with the application of DNA metabarcoding to survey the proportional species richness of insects in Denmark. We do so by testing whether the proportional richness of insects detected with the methodology is equal to the proportions registered in the Danish species database. Furthermore, we examine patterns in insect richness and diversity across biogeographic regions and dominant land covers, and use hoverflies (Syrphidae) as a case taxon to detect patterns in large-scale species distributions. Finally, we examine whether we detect new species occurrences for the country and species in the Danish Red List.

## 2. Methods

**Figure 1.**
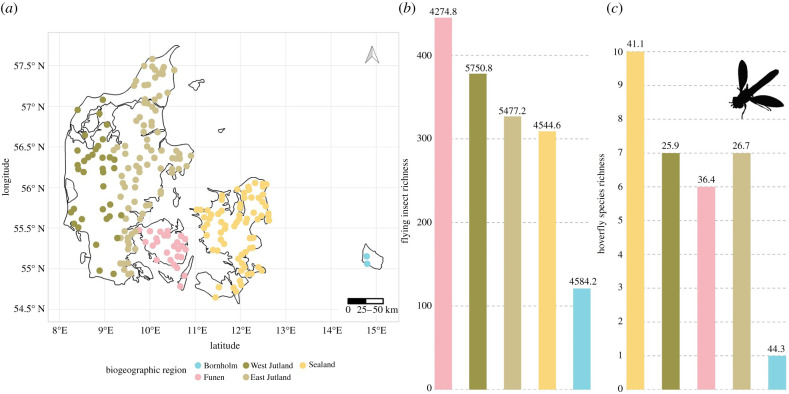
Observed total insect richness and hoverfly richness across biogeographic regions in Denmark. Bornholm is included for visualization but excluded from the models due to low sample sizes. (*a*) Biogeographic regions separated by main islands and the last glacial ice sheet separating the Island of Jutland in two regions. (*b*) Observed flying insect richness across regions and (*c*) hoverfly richness across regions. Colours represent unique biogeographic regions and numbers above bars represent mean Chao1 estimated diversity.

Car net sampling was carried out by volunteers along 5 km manually designed routes during June 2018 in Denmark (figure 1*a*). To cover activity periods and sites of as many species as possible, sampling was carried out within the time intervals 12.00–15.00 and 17.00–20.00, and routes were placed in forest, urban areas, farmland, wetland and grassland. Each route was driven from start to end and then back to the start again at a maximum speed of 50 km h^−1^. Bulk insect samples were placed in 96% ethanol and stored in a −20°C chest freezer prior to DNA extraction. Further information on the car net design, citizen science sampling, route design and environmental variables can be found in Svenningsen *et al.* [25] and electronic supplementary material, SI.

DNA extraction, qPCR, PCR, library building and sequencing were carried out on 365 samples from 218 routes. The full protocol from sample processing to library build can be obtained here: https://dx.doi.org/10.17504/protocols.io.bmunk6ve. Data from the fwh primer pair were used in this study. Further information on the laboratory methods can be found in electronic supplementary material, SII.

### (a) Bioinformatics analysis

Sequencing libraries were demultiplexed using cutadapt (v. 1.11) [26]. Amplicon sequence variants (ASVs) were identified and chimeras removed with DADA2 [27]. Redundant sequences were removed with the LULU algorithm [28]. The sequence ID tool from GBIF (https://www.gbif.org/tools/sequence-id) was used for taxonomic assignment. Further information on the bioinformatics analysis and sequencing results can be found in electronic supplementary material, SIII.

All statistical analyses were carried out in RStudio (v. 3.6.1). To focus the analysis on species occurrences, the ASV table was converted to presence/absence prior to analysis; however, for diversity assessments, the sample read counts were rarefied to the minimum sample read count (8890 reads per sample) prior to diversity estimates and indices were calculated. Rarefaction curves for the raw data can be found in electronic supplementary material, SIII.

### (b) Statistical analysis

**Figure 2.**
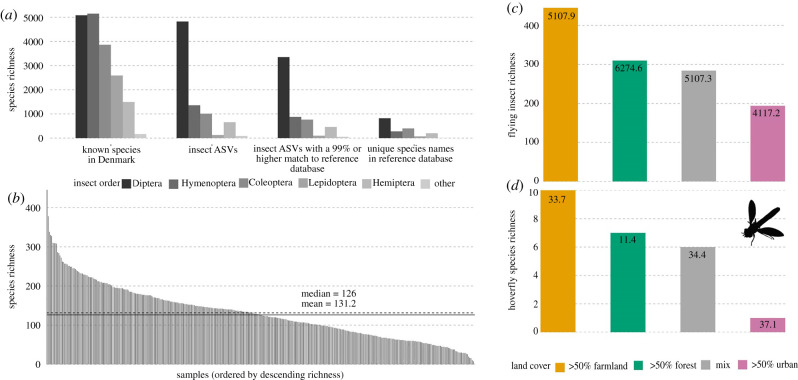
ASV/species richness in (*a*) the five largest insect orders and all small orders (other) in the Danish species database (*known species in Denmark*) compared to insect *ASVs* (may contain multiple unique ASVs for each named species), ASVs with a good match (*insect ASVs with a greater than or equal to 99% match to reference database*) and *unique species names in the reference database* (without multiple ASVs assigned to the same species). (*b*) Median (solid line) and mean (dotted line) species richness per sample in descending order, the *x*-axis represent each sample and the *y*-axis is the observed ASV richness. (*c*) Observed ASV richness across land covers, each colour indicates a land cover type (*x*-axis). (*d*) Observed hoverfly ASV richness across land cover types. Numbers on bars represent the average Chao1 estimated diversity.

The proportion of insect species in each insect order was compared to the Danish species list (www.allearter.dk), excluding the non-flying orders Phthiraptera, Siphonaptera, Zygentoma and Microcoryphia. We based the proportional comparison on unique BINs (thus excluding sequence variants, *n* = 4653) within each insect order filtered by match to class Insecta and included comparisons to a greater than or equal to 99% reference match and unique species names for visualization of differences in sequence and taxonomic richness estimates (figure 2).

We used a two proportions *z*-test, to test differences in proportion of ASVs within insect order, family, genus and species, and amount of species detected within each insect order, between our results and the species in the Danish species database. We examined insect and hoverfly richness and diversity across biogeographic regions, excluding the island of Bornholm due to low sample size (figure 1*a*), and a greater than or equal to 50% or a mixed land cover (as a categorical variable) with mixed-effects models (lmer function in the lme4 package) [29]. Model selection was carried out with the dredge function from the MuMIn package [30]. ASV richness was used as a richness metric and Chao1 estimate as a diversity metric.

Furthermore, we investigated whether the car net samples contained species not registered in the public Danish species database or in the subset of data from the Global Biodiversity Information Facility from Denmark [31] and neighbouring countries Sweden, Norway and Germany [32]. For reference database comparison, we based final taxonomic assignments on a greater than or equal to 99% match across the entire query sequence and filtered based on unique species names. Species from our results without occurrences in Denmark or neighbouring countries were manually checked for occurrences in each country in Fauna Europaea [33], and BOLD systems v. 4 [34]. Lastly, we examined if we detected species registered in the Danish Red List and their status. To examine sample coverage, we generated species accumulation curves and additional richness estimates; see electronic supplementary material, SIV for methods and results.

## 3. Results

From 365 car net samples, we identified 15 insect orders, 240 families, 1273 genera and 2114 species. This corresponds to equal proportions of flying insect orders, half of all families, a quarter of all genera and 11.3% of all species, on the complete Danish species list (electronic supplementary material, table S1; figure 2*a*).

The median sample richness was 126 unique ASVs per sample (figure 2*b*). Diptera was the most species rich group, followed by Hymenoptera, Coleoptera and Hemiptera, while Lepidoptera and Trichoptera were less represented in our samples compared to Danish estimates. The remaining insect orders were all represented by a proportion of less than 2% (table 1, figure 2*a*). Equal proportions to the known richness were obtained for Hemiptera, Orthoptera, Mecoptera, Dermaptera, Odonata, Plecoptera and Ephemeroptera (table 1). We did not detect any members of the flying insect orders Dictyoptera, Megaloptera, Raphidioptera and Strepsiptera.

**Table 1.** Results of the two proportion z-test results on the proportion of species in each insect order and the proportion of species detected proportionally to known insect species for Denmark. Each insect order was tested against the known species number for Denmark within the insect order to assess whether our method detected equal or unequal species proportions. Significant and unequal proportions are marked with italics.

insect order	Pearson's *X*^2^	*p*-value	lower CI	upper CI	relative proportion of individual ASVs in car nets (%)	relative proportion of individual species Denmark (%)	proportion of species detected in car nets compared to species in Denmark (%)
*Diptera*	*1242.1*	<*0.001*	*0.25*	*0.29*	*54.1*	*27.1*	49.5
*Hymenoptera*	*89.63*	<*0.001*	*−0.08*	*−0.05*	*20.6*	*27.4*	18.6
*Coleoptera*	*169.68*	<*0.0001*	*−0.1*	*−0.07*	*12.2*	*20.6*	14.7
*Lepidoptera*	*487.88*	<*0.001*	*−0.12*	*−0.11*	*2.2*	*13.8*	4
Hemiptera	1.84	0.17	−0.003	0.015	8.6	8	26.7
*Trichoptera*	*26.1*	<*0.001*	*−0.01*	*−0.01*	*0.17*	*0.92*	4.7
*Thysanoptera*	*4.71*	*0.03*	*−0.0001*	*0.006*	*0.9*	*0.6*	37.2
*Neuroptera*	*10.25*	*0.001*	*−0.004*	*−0.002*	*0.04*	*0.3*	3.2
*Psocoptera*	*8.67*	*0.003*	*0.001*	*0.005*	*0.64*	*0.33*	48.4
Odonata	3.16	0.08	−0.003	−0.0002	0.15	0.32	11.7
Orthoptera	0.71	0.4	−0.002	0.001	0.13	0.2	15.8
Ephemeroptera	0.33	0.57	−0.002	0.001	0.002	0.002	18.6
Plecoptera	3.24	0.07	−0.002	−0.003	0.02	0.13	4
Dermaptera	<0.00^a^	1	−0.001	0.0005	0.02	0.03	16.7
Mecoptera	0.10^a^	0.76	−0.001	0.001	0.04	0.02	50

^a^Pearson's *χ*^2^ values were uncertain, due to the low number of taxa.

Insect richness was on average lower in Sealand, although variation in richness could not significantly be explained by biogeographic regions (electronic supplementary material, SV). However, diversity was significantly lower in Sealand compared to Jutland (figure 1*b*; electronic supplementary material, SV). Lower insect richness and diversity was significantly associated with a greater than 50% urban land cover (figure 2*c*; electronic supplementary material, SV). We did not detect any significant variation in hoverfly richness across biogeographic regions or land covers; however, hoverfly diversity was high in Sealand compared to Jutland, and within Jutland, diversity was higher in the western part of the peninsula (figure 1*c*; electronic supplementary material, SIV). Hoverfly diversity was on average lowest in greater than 50% forest land cover, however, this pattern was only marginally significant or a trend.

We detected 319 species not registered in Denmark (electronic supplementary material, table S4). The majority of the species had occurrences in the neighbouring countries leaving 17 new species for the region (electronic supplementary material, table S5). Of the 1829 unique species detected, 1440 species (79%) were found in the Danish Red List and the majority were not evaluated (NE) (88%). One species was registered as vulnerable (VU), *Bruchus rufimanus* (broadbean weevil), though notably it is a crop pest, and three species (two bees and a hoverfly) were registered as near threatened (NT) (electronic supplementary material, table S7).

## 4. Discussion

Using car nets and DNA metabarcoding, we detected insect orders in proportion to their richness in the Danish species database (15 insect orders out of 19 known), including almost half of all flying insect families and 17 species with no occurrences from Denmark or neighbouring countries. We detected patterns in richness and diversity across biogeographic regions and dominant land covers. However, with more informative explanatory variables, e.g. pollen data, farmland-specific variables, e.g. pesticide use and crop type, and proportional land cover [25], further trends may be detected with a higher degree of explained variation.

The insect orders Dictyoptera, Megaloptera, Raphidioptera and Strepsiptera were not detected in the car nets. However, species of Dictyoptera and Raphidioptera only take flight occasionally and usually stay close to vegetation [35,36], Megaloptera species tend to stay close to water bodies [36] and Strepsiptera females are endoparasites of insects and only the very short-lived males take flight [37]. Since we sampled in the month of June 2018, we did not detect species that are present before and after June, and a longer sampling season will most likely increase the amount of species detected with our methodology.

For several years, the Swedish Malaise Trap Project (SMTP) has collected and morphologically identified flying insects [38]. Similar to our findings based on DNA, they found Diptera to be the most species rich group (75%), followed by Hymenoptera (15%), with less than 10% of the total catches belonging to the other insect orders and Acari. In our samples, we detected higher proportions of Hemiptera and Coleoptera, compared to SMTP. Although our results are based on one month's sampling in 2018, it is strikingly similar in overall taxonomic composition to the multiyear national assessment for Sweden based on Malaise traps. Similar to SMTP, we also find most of the new species detected belong to Diptera and Hymenoptera [39]. However, our approach is much quicker and covers a much larger geographical area. Car nets can sample over 1000 individuals per sampling trip (approx. 10–20 min) under favourable conditions which is similar to 24 h of sampling with Malaise traps [6].

The DNA metabarcoding approach as employed here, or employed in concert with other sampling approaches, has definite advantages. However, given existing reference databases, it also has limits. First, several methodological steps in the laboratory inevitably introduce biases all the way from DNA extraction to sequencing, which have been well discussed elsewhere. Second, processed sequences need to be assigned to taxa (ASVs), the details of which depend upon algorithmic rules, which can influence the number of species identified as well as their boundaries. Finally, if ASVs are to be matched to morphologically named species (which they do not necessarily have to be for community and diversity assessments), DNA metabarcoding relies on updated reference databases and so is only as good as those databases. The databases for Denmark are relatively complete, but for many countries, particularly biodiverse countries, they are not.

Even in well-studied regions, reference databases differ among taxa. For example, Diptera and Hymenoptera have relatively fewer species references in BOLD compared to other insect orders, and small-bodied taxa are especially underrepresented [40]. Since car nets catch large numbers of small Dipterans, DNA barcoding could in the future be used to generate references and fill the reference library gap for small-sized Diptera as well as other unknown taxa. The non-destructive DNA extraction method used in this study further has the advantage that single individuals can be isolated, morphologically identified and re-analysed to generate reference sequences.

One caveat with car nets as a monitoring tool is that certain weather conditions have to be present for sampling to be carried out, i.e. no rain and low wind speed, which makes it difficult to use in some areas that are prone to high wind speed, e.g. coastal areas. Another caveat is that since sampling is carried out using cars, roads have to be present and in good condition. Driving speed and the number of stops may have an impact on how many insects are sampled [25] and, therefore, requires routes to be designed with considerations of stops, turns and road conditions. For example, urban areas have a higher number of stop signs, road crossings etc. compared to rural areas, which in turn more often have gravel roads that require the car to drive slower than on a paved road.

Nonetheless, for many uses, the limits and caveats associated with the combination of car nets and metabarcoding are outweighed by the benefits. By designing a simple, standardized citizen science project, we were able to sample at a large spatial scale within one month, with a response rate of more than 75% of the projected samples returned. As such, car net sampling with the help of citizen scientists could be a promising tool for monitoring flying insects across time and space.

## 4. Conclusion

Car net sampling combined with DNA metabarcoding may be a useful tool for monitoring flying insects across spatial and temporal scales. Furthermore, car nets can detect unregistered species, be applied to monitor selected taxa, e.g. hoverflies and mosquitoes, and have the potential application as a large-scale monitoring method.

## Data accessibility

Data used for analysis in this manuscript are included as electronic supplementary material. Scripts used for analysis can be accessed at: https://github.com/CecSve/InsectMobile_CarNet.
